# Metachronous Sporadic Desmoid Tumors Arisen in the Stomach and the Cecum

**DOI:** 10.7759/cureus.14847

**Published:** 2021-05-04

**Authors:** Ichiro Tamaki, Hidenori Takahara

**Affiliations:** 1 Department of Surgery, Ako City Hospital, Ako City, JPN

**Keywords:** desmoid-type fibromatosis, sporadic, metachronous desmoid tumor, intraabdominal desmoid-type fibromatosis, laparoscopic surgery

## Abstract

Desmoid-type fibromatosis (DF) is a rare soft-tissue tumor demonstrating fibroblastic to myofibroblastic differentiation, recognized as a biologically intermediate, locally aggressive tumor; however, it can be clinically lethal due to its infiltrative growth and risk of locoregional recurrence. Desmoid-type fibromatoses can arise from any part of the body, however, intra-abdominal DFs comprise only 8% of all DFs. We report a case of a male in his 60s who presented with the metachronous occurrence of DF: gastric DF followed by cecal DF with two years of clinical interval. The latter tumor (cecal DF) developed under scheduled postoperative surveillance of laparoscopic gastrectomy. Although a surgical wound is known to be an inductive factor for DFs, the cecal DF developed in a part that was not a surgical site in the previous operation. Curative resection is the first treatment option when the tumor shows progression in size. Following the curative resection, close observation should be provided because of the risk of locoregional recurrence.

## Introduction

Desmoid tumors, also recognized as desmoid-type fibromatosis (DF), are very rare soft-tissue tumors. Desmoid-type fibromatosis is classified as “intermediate, locally aggressive” tumor in the WHO classification of soft tissue tumors [1]. The incidence is known as 2.4 to 4.3 per 1 million per year [2]. Desmoid-type fibromatoses are classified into three types according to their original site: extra-abdominal, abdominal-wall, and intra-abdominal types, and their occupational rate is 43%, 49%, and 8%, respectively [2]. Desmoid-type fibromatoses arise sporadically or in association with familial adenomatous polyposis syndrome (FAP), a complex genomic syndrome caused by a germline mutation in the adenomatous polyposis coli (APC) gene [3, 4]. Desmoid-type fibromatosis is recognized as a biologically intermediate neoplasm, rarely metastasizing; however, its high risk of local recurrence and extensive resection leads to certain risks of mortality [5].

Here, we represent a case of sporadic, metachronous digestive tract DFs: gastric wall and cecal wall with two years of time interval.

## Case presentation

History of illness

**Figure 1:**
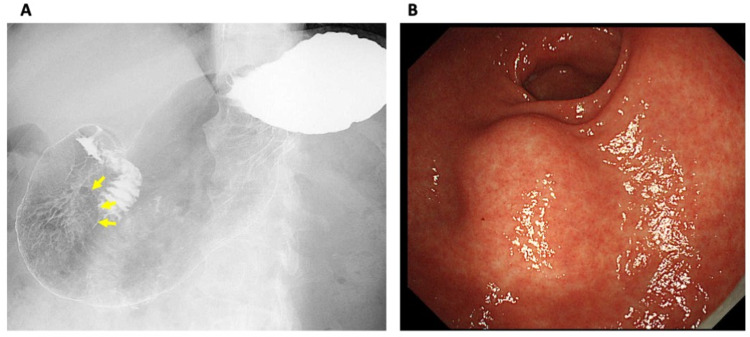
Gastric contrast examination and gastrointestinal endoscopy A: Gastric contrast examination shows a smoothly circumscribed mass in the lower part of the stomach (arrows). B: Gastrointestinal endoscopy shows an intraluminal soft tumor in the lower part of the stomach.

A man in his 60s was admitted to our hospital for the evaluation of a gastric abnormality which was found by a gastric contrast examination in the annual health checkup (Figure 1A). He had no obvious symptoms correlated with the digestive tract. He had a medical history of surgery for cervical disc herniation in his 50s, and cholecystectomy for cholecystitis three years before the admission. Gastrointestinal endoscopy performed in advance of the cholecystectomy pointed no abnormal findings at that time. He had no remarkable family history, including colonic polyp or cancer.

Clinical investigation (gastric tumor)

**Figure 2:**
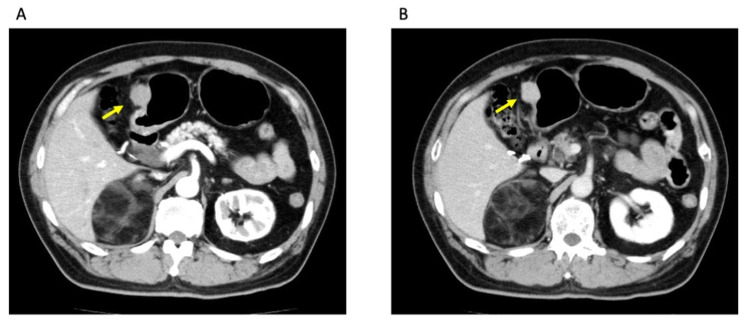
Contrast-enhanced CT (CECT) Contrast-enhanced CT (CECT): A: early phase, B: late phase. CECT shows a solid tumor with mild vascularity in the stomach wall (arrows).

Gastrointestinal endoscopy revealed a submucosal tumor around 30 mm in diameter in the lower part of the stomach (Figure 1B). Endoscopic ultrasonography (EUS) exhibited a round and echoic heterogeneous tumor, which developed from the proper muscle layer. Contrast-enhanced CT (CECT) represented a tumor with intermediate vascularity, 25 mm in diameter, protruding from the stomach wall upon free space (Figure 2). Colonoscopy, performed as a screening examination, found no abnormality in the lower digestive tract. The first clinical diagnosis was a gastrointestinal stromal tumor (GIST) in the stomach, although EUS-guided biopsy was not performed. Curative resection was intended as the first treatment option.

Treatment and pathological findings (the first surgery)

**Figure 3:**
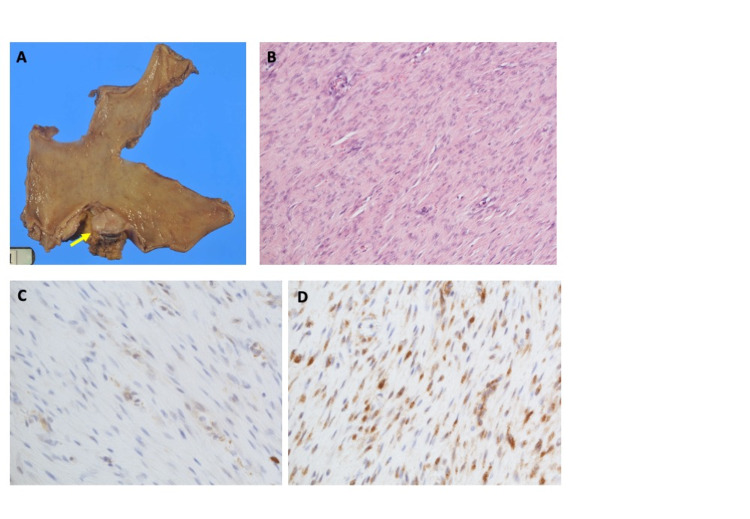
Pathological findings A: Gross examination of the resected specimen. A submucosal solitary tumor 25 mm in the maximum diameter is seen (arrow). B: Hematoxylin-eosin (HE) staining of the specimen (x20). HE stained specimen represents bland spindle cells with faintly eosinophilic cytoplasm in a syncytial pattern separated by dense collagen bundles. C: Immunohistochemistry (IHC) of α-SMA (×40). α-SMA represents faint staining in the specimen. SMA: smooth muscle actin. D: Immunohistochemistry (IHC) of β-catenin (×40). Nuclear immunoreactivity for β catenin is shown in the specimen.

Laparoscopic distal gastrectomy was performed for curative resection. Gross examination of the resected specimen exhibited a submucosal solitary tumor of the stomach 25 mm in the maximum diameter (Figure 3A). Harvested regional lymph nodes revealed no lymph node metastases. The tumor division surface represented a solid tumor developed in the proper muscle layer.

Hematoxylin-eosin (HE) staining of the specimen represented bland spindle cells with faintly eosinophilic cytoplasm in a syncytial pattern. These cells are arranged between dense bundles of collagen (Figure 3B). Mitosis rate and MIB-1 index were 4/50 high power field (HPF) and 2%, respectively. Immunohistochemistry (IHC) demonstrated negative for c-kit, CD34, S-100, and desmin. Additionally, DOG1 was found to be negative and α-SMA (smooth muscle actin) represented faint staining (Figure 3C). At that time, our tentative diagnosis was leiomyoma in the stomach wall. Although the diagnosis was not final, the patient did not request further pathological study for the differential diagnosis of the tumor that was resected curatively. 

The postoperative course was uneventful and careful postoperative follow-up was started.

Clinical investigation (tumor of the right colon)

**Figure 4:**
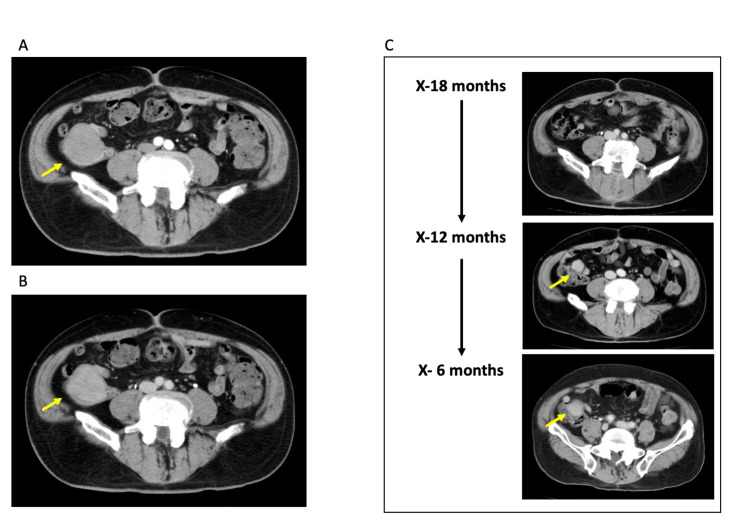
Contrast-enhanced CT (CECT) A, B: Contrast-enhanced CT (CECT) (A: early phase, B: late phase). CECT shows a dense tumor in the ileocecal area. The tumor represents mild vascularity. C: Chronological alteration of the ileocecal tumor. The time point X stands for the date when the ileocecal tumor was noticed. Retrospectively the tumor was evident at the time point X-12 months. The tumor showed an expansive growth retrospectively.

Scheduled CECT was performed every six months together with blood analysis as a regular checkup after the surgery. Two years after the gastrectomy, CECT revealed a solid tumor in the ileocecal area 50 mm in diameter representing intermediate vascularity (X year, Figures 4A, 4B). We reviewed the prior CECT studies retrospectively, then a solid nodule with 15 mm in the maximum diameter was seen in the cecal wall (X-1 year), suggesting gradual growth of the tumor (Figure 4C).

**Figure 5:**
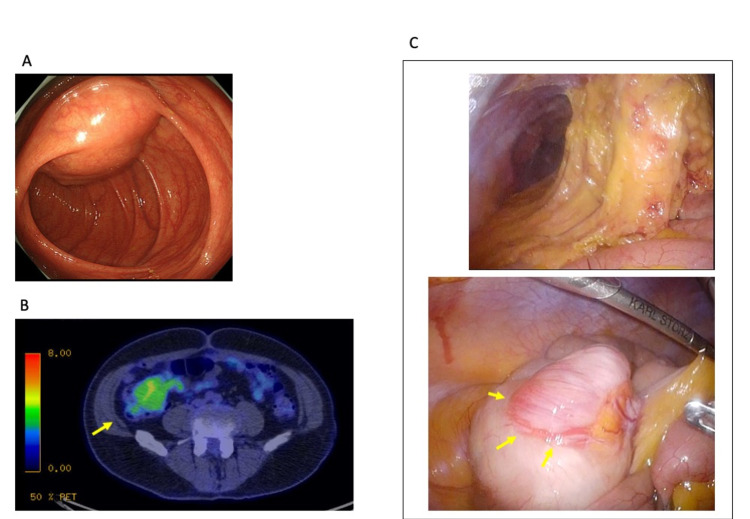
Colonoscopy/18F-FDG positron-emission tomography-CT/laparoscopic findings A: Colonoscopy. Total colonoscopy found a submucosal tumor with cushion sign in the cecam. B: 18F-FDG Positron-emission tomography (PET) CT. 18F-FDG PET-CT showed a mild accumulation of the 18F-FDG in the tumor site (SUVmax = 5.0) (arrow). Distant metastasis was not evident in the study. C: Intraoperative pictures (laparoscopic pictures). Laparoscopically, intraabdominal adhesion was seen mostly upper part of the abdominal cavity due to prior surgery (laparoscopic distal gastrectomy). In contrast, intraabdominal adhesion was not seen around the ileocecal parts. The tumor protruding from the cecal wall was seen (arrows).

Colonoscopy showed a submucosal tumor in the cecal wall (Figure 5A). 18F-FDG Positron-emission tomography (PET) -CT showed a mild accumulation of the 18F-FDG in the tumor site (SUVmax = 5.0) (Figure 5B), whereas distant metastasis was not evident. The preliminary diagnosis included GIST, neuroendocrine tumors, and leiomyoma. Elective surgery was scheduled for curative resection of the tumor as the first treatment option.

Treatment and pathological findings (the second surgery)

Laparoscopic ileocecal resection was performed for curative resection. Laparoscopically, intraabdominal adhesion due to the prior surgery was found only in the upper abdominal cavity, whereas no anatomical alteration nor adhesion made by the former surgery was seen around the ileocecal part. The tumor was located laparoscopically easily, representing protrusion from the cecal wall (Figure 5C).

**Figure 6:**
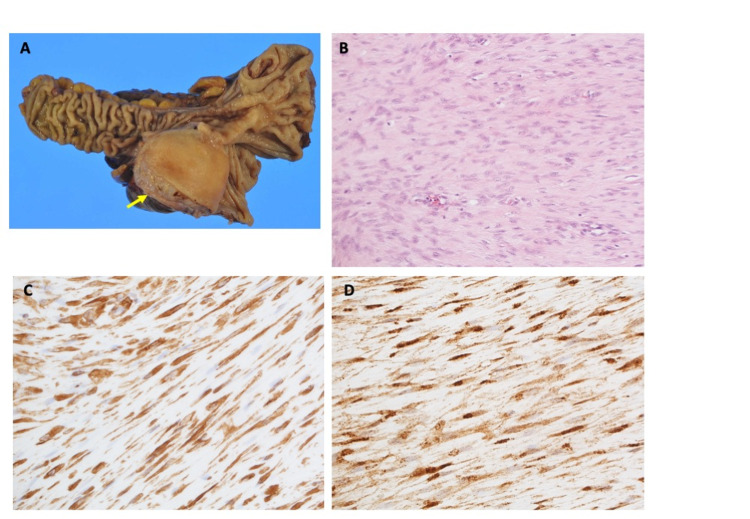
Pathological findings A: Gross examination of the divided specimen. A solid tumor with 55 mm in the maximum diameter protruding from the cecal wall is shown (arrow). B: Hematoxylin-eosin (HE) staining of the specimen (×20). HE stained specimen represents bland spindle cells with faintly eosinophilic cytoplasm in a syncytial pattern separated by dense collagen bundles. C: Immunohistochemistry (IHC) of vimentin (×40). The specimen is positive for vimentin. D: Immunohistochemistry (IHC) of β-catenin (×40). Nuclear immunoreactivity for β-catenin is shown in the specimen.

Gross examination of the divided specimen revealed a solid tumor with 55 mm in the maximum diameter (Figure 6A). Hematoxylin-eosin (HE)-stained specimen represented nodular clusters of spindle cells containing eosinophilic syncytial cytoplasm arranged between dense collagen bundles (Figure 6B). The mitosis rate was 4/50 HPF. Immunohistochemistry exhibited negative for c-kit, S-100, CD34, DOG-1, and desmin. Lastly, the following IHC findings were achieved: positive for α-SMA (partially), vimentin (Figure 6C), and nuclear reactivity for β-catenin (Figure 6D). These findings lead to the final diagnosis of desmoid-type fibromatosis of the cecum.

The specimen of the gastric submucosal tumor resected two years before the second surgery was undergone IHC study retrospectively. It exhibited positive for β-catenin (Figure 3D), whereas negative for caldesmon, representing that the gastric tumor was desmoid, not leiomyoma.

The postoperative clinical course of the second surgery was also uneventful, and the patient is under close observation.

## Discussion

Desmoid-type fibromatosis is a very rare soft-tissue tumor demonstrating fibroblastic to myofibroblastic differentiation, recognized as biologically intermediate [1], however, it can be clinically lethal due to its infiltrative growth and risk of locoregional recurrence [5, 6]. As aforementioned, intra-abdominal DFs are quite rare, and the incidence can be estimated to approximately less than 1 per million per year [2].

The presenting case is a man in his 60s, although there is a 2- to 3.5-fold increased incidence in women [7], and the mean age is reported to be 41.6 years [4]. We considered the differential diagnosis of Gardner’s syndrome or FAP for him, however, a total colonoscopy revealed no colon polyps, excluding the possibility of FAP (unless appropriate treatment including prophylactic colectomy, patients of FAP have an almost 100% risk of developing colorectal cancer) [3]. The incidence of DFs for the case should be concluded as sporadic. 

As mentioned in the Case Presentation section, we did not reach a diagnosis of DF immediately. For the diagnosis of the gastric submucosal tumor resected in the first surgery, an IHC study including c-kit, CD34 (a marker for GISTs), S-100 (a marker for neurogenic tumors), and desmin (a marker for leiomyoma) was performed and turned to be negative. The result was confusing for us at that time, then additional faint staining of α-SMA lead to the tentative diagnosis of gastric leiomyoma. As for the submucosal tumor of the cecal wall resected in the second surgery, c-kit, CD34, DOG-1, S-100, and desmin were negative. Additionally, α-SMA exhibited to be positive faintly, finally β-catenin was found to be positive and lead to the final diagnosis of DF of the cecal wall [6]. Positivity for vimentin supports the diagnosis [6]. Retrospectively, β-catenin positivity was also recognized in the resected specimen of the gastric submucosal tumor, which then leads to the diagnosis of gastric DFs. The incidence of DFs is very rare, so the pathologist of the hospital made a consultation about the specimens with the Japanese Society of Pathology Expert Committee, and finally confirmed both specimens to be DFs pathologically. 

Desmoid-type fibromatosis is recognized to have locoregional recurrence sometimes, and the relative risks become greater when the prior surgery had positive resection margins or residual tumor [8]. In addition to this, surgical trauma, including abdominal surgeries, has been linked to the development of desmoid tumors in prior surgical areas. This phenomenon is adapted in patients with FAP or sporadic DFs [5]. According to Church, surgical manipulation or tension upon organ soft tissue is responsible for DFs occurrence on DFs-prone people [5]. Devata et.al. mentioned the underlying mechanism: dysregulation of β-catenin either through the APC gene (FAP-related DFs) or CTNNB1 gene mutations (sporadic DFs) under the wound healing process leads to the occurrence of DFs [6]. Conclusively, mesenchymal stromal cell mutations and wound healing contribute to the etiology of desmoid tumors [9]. As to the presenting case, DFs in the cecal wall occurred following laparoscopic distal gastrectomy. Here, one doubt arises: ileocecal parts, in which the tumor emerged is not a part that had surgical manipulation in former laparoscopic gastrectomy. The laparoscopic findings showing no adhesive alteration around the cecal submucosal tumor support the discrepancy. Regarding this point, it is safe to say the presenting case is a metachronous DFs in the stomach and the cecum. 

The case presentation has limitations as follows. Firstly, the aforementioned somatic gene mutation of the specimen, CTNNB1 is not evaluated for the case although 85% of sporadic DFs harbor the mutation [10]. As for possible germline mutations underlying DFs occurrence, it is not evaluated. However, family aggregation is not evident in the case.

Regarding post-surgical follow-up for the patients, close follow-up including CECT study every six months and blood analysis every three months is ongoing.

## Conclusions

Desmoid-type fibromatosis, especially intra-abdominal DF, is a very rare disease. However, the differential diagnosis should include DFs for a submucosal tumor exhibiting spindle shape cells that are negative for c-kit, S-100, and desmin. Curative resection is the first treatment option when the tumor shows progression in size. Following the curative resection, close observation should be provided because of the risk of locoregional recurrence. 
